# Data Analytics Applications for Streaming Data From Social Media: What to Predict?

**DOI:** 10.3389/fdata.2018.00002

**Published:** 2018-09-11

**Authors:** Frank Emmert-Streib, Olli P. Yli-Harja, Matthias Dehmer

**Affiliations:** ^1^Predictive Medicine and Data Analytics Lab, Department of Signal Processing, Tampere University of Technology, Tampere, Finland; ^2^Institute of Biosciences and Medical Technology, Tampere, Finland; ^3^Institute for Systems Biology, Seattle, WA, United States; ^4^Department for Biomedical Computer Science and Mechatronics, UMIT - The Health and Lifesciences University, Hall in Tyrol, Austria; ^5^Faculty for Management, Institute for Intelligent Production, University of Applied Sciences Upper Austria, Steyr, Austria; ^6^College of Computer and Control Engineering, Nankai University, Tianjin, China

**Keywords:** social media, data analytics, prediction model, forecasting, big data, computational social science, scientometrics, data science

## Abstract

Social media in general provide great opportunities for mining massive amounts of text, image, and video-based data. However, what questions can be addressed from analyzing such data? In this review, we are focusing on microblogging services and discuss applications of streaming data from the scientific literature. We will focus on text-based approaches because they represent by far the largest cohort of studies and we present a taxonomy of studied problems.

## 1. Introduction

The establishment of the World Wide Web (WWW) in the 1990s revolutionized the communication between people in many different and profound ways affecting our professional and social life alike. One particular consequence of the WWW has been the creation of social media that provide a forum for the direct exchange of digital information in the form of texts, photos, or videos, e.g., via blogs, microblogs, photo sharing, video sharing, social bookmarking, virtual worlds, social gaming, or social networking web pages. The top sites such as Twitter, Facebook, LinkedIn, and Google+ are used by hundreds of millions of active users worldwide. In the following, we will focus on text-based social networking services for microblogging that are publicly accessible. This excludes Instagram (image-based) and Youtube (video-based) but also Whatsapp (not publicly accessible chats) from our considerations.

Due to the relatively brief history of the WWW and the social networking services there is still a severe lack of understanding what, e.g., the information provided by microblogs can be used for. For this reason, we provide a review of the literature with a focus on application areas of prediction models that have been developed so far for analyzing data from microblogging services.

By prediction models we mean methods that aim at forecasting new events rather than merely summarizing or describing information contained in data. For instance, among the first studied questions of social media were investigations related to the topological structure of social networks. Specifically, the degree distribution, the community structure and motifs of acquaintance networks representing the “friendships” among members of social networking services, corresponding to nodes in such graphs, have been investigated (Java et al., 2007; Aparicio et al., 2015). Such studies are more descriptive in nature. Instead, in this review we present an overview of the literature that use social media data for classification, regression, or time series prediction problems.

## 2. General application fields and number of publications

We are starting our review my demonstrating that the field of social media analytics is of great interdisciplinary interest occupying already today a large share in the literature.

In order to show this, we are using the Web of Science (WoS) (Clarivate Analytics, 2009) database, which is an online subscription-based citation indexing service operated by Clarivate Analytics. WoS contains comprehensive information about published scientific articles in all areas. We used WoS searching for articles containing the name of a microblog either in the title, abstract, or as a keyword we found: Twitter: 16614, Facebook: 15483, Tumblr: 175, GNU social (previously known as StatusNet and Laconica): 72, Plurk: 56. From this we conclude that the by far most frequently investigated microblogs in the literature are Twitter and Facebook. For this reason, we will focus on these in the following.

**Figure 1:**
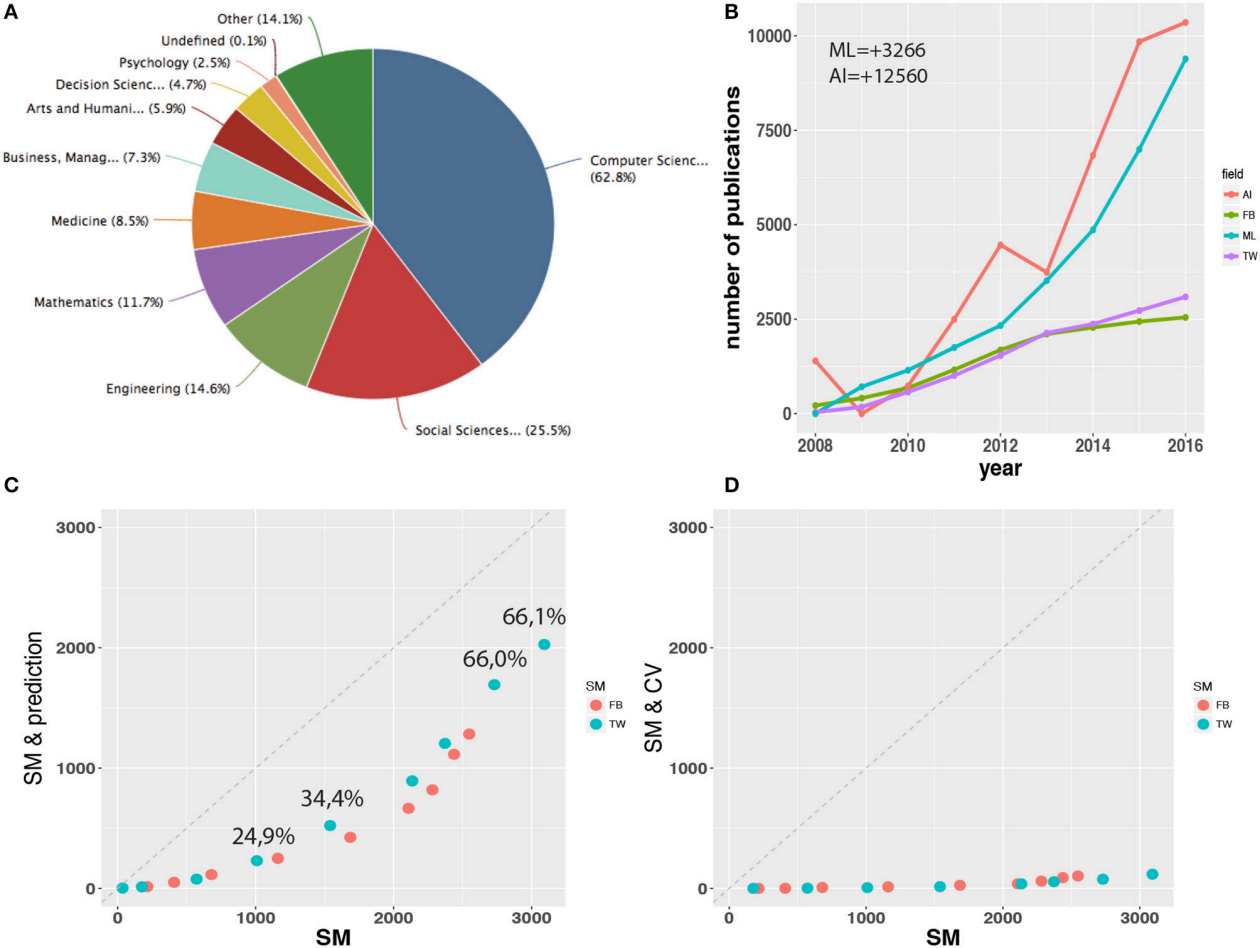
**(A)** Scientific fields of published articles investigating Twitter (TW) or Facebook (FB). **(B)** The number of published articles containing the keywords Twitter, Facebook, “machine learning” (ML) or “artificial intelligence” (AI). The numbers ML = +3266 and AI = +12560 indicate the baseline shift for ML and AI. **(C)** Scatter plot comparing articles containing the social media (SM) keyword Twitter or Facebook with articles containing additionally “prediction” or “forecast.” The shown “forecast.” The shown percentages are for Twitter giving the fraction of prediction related publications referred to all publications. **(D)** Similar to **(C)**, but now containing additionally the keywords “cross validation” (CV) or “resampling”.

In Figure 1A. an overview of scientific fields is shown as tagged to published articles containing the keyword Twitter or Facebook, either in the title, the abstract, or as a keyword. It is not surprising that most publications are computer science or social science related. However, also quite a large fraction of papers comes from medicine, management & business, and even arts & humanities. Interestingly, the fraction of psychology related publications is rather low despite the fact that intuitively one would name this field first due to the personal nature of tweets and Facebook postings. One reason for this underrepresentation may be related to computational obstacles psychologists need to overcome when they want to analyze social media data because available tools may not allow to tackle targeted research questions as conceived by psychologists.

In Figure 1B, we show the number of published articles containing the keywords Twitter, Facebook, “machine learning” or “artificial intelligence.” For papers containing the words Twitter or Facebook these numbers are total numbers, for “machine learning” and “artificial intelligence” these numbers are subtracted by the minimal number of published papers in these fields between 2006 and 2016. For “machine learning” this number is 3266 and for “artificial intelligence” it is 12560. By subtracting these numbers we shifted both curves downward (baseline shift) to make all four curves comparable with each other due to the fact that articles investigating Twitter or Facebook commenced only around 2008 whereas the work in machine learning and artificial intelligence goes much further back. In this sense, the curves shown for machine learning and artificial intelligence provide only information about *new research directions* as started around 2008. From this comparison we learn that the proportion of social media related publications compared to all articles involving machine learning or artificial intelligence is amazingly high, making it about 1/4 in 2016. Another tendency we can observe is that the number of Twitter related publications is overtaking Facebook since 2013. We did not include the years 2017 and 2018 in Figure 1B. because the counts in WoS are still incomplete but also for these years we find this trend to continue (data not shown).

## 3. Applications

### 3.1. Specific scientific application fields

The idea of utilizing data from social media for making predictions has generated great interest (Kalampokis et al., 2013; Schoen et al., 2013). The question is what can one predict based on such data? Prominent examples for such studies are prediction models that investigated the emotional constitution of people (Fernandez et al., 2012; Kross et al., 2013; Ortigosa et al., 2014), personal traits and characters (Kosinski et al., 2013), stock market behavior (Bollen et al., 2011; Siganos et al., 2014), election results (Alonso and Vilares, 2016; Tumasjan et al., 2011).

Further examples are consumer behavior (Ringelhan et al., 2015), public health (Sinnenberg et al., 2017), opinion flow (Wu et al., 2014), sharing cascades (Kupavskii et al., 2012; Cheng et al., 2014), account classification (Chu et al., 2010, 2012; Dickerson et al., 2014), conflicts among friends (Liu and Weber, 2014), demographics of users (Culotta et al., 2015), mental health (Guntuku et al., 2017), heart disease (Eichstaedt et al., 2015), tourism (information search and decision-making behaviors) (Zeng and Gerritsen, 2014), word-of-mouth (WOM) or consumer reviews (Zhang et al., 2012), box-office revenue of movies (Asur and Huberman, 2010), levels of rainfall (Lampos and Cristianini, 2012), earthquakes (Sakaki et al., 2010), theoretical implications introduced by social media (Kane et al., 2014). In Table 1 we provide a comprehensive overview of many important questions that have been studied using social media data. We would like to note that here we emphasized the “What to predict” aspect of these studies by highlighting the questions that have been addressed.

**Table 1:** An overview of questions addressing “What do predict” with social media data.

**“What to predict”**	**References**
Bot detection (account classification)	Chu et al. (2010, 2012); Dickerson et al. (2014)
Box-office revenue of movies	Asur and Huberman (2010)
Company value	Luo and Zhang (2013)
Conflicts among friends	Liu and Weber (2014)
Consumer behavior	Ringelhan et al. (2015)
Crime incidents	Gerber (2014); Aghababaei and Makrehchi (2016)
Demographics of users	Culotta et al. (2015)
Earthquakes	Sakaki et al. (2010)
Election results	Alonso and Vilares (2016); Tumasjan et al. (2011)
Emotional constitution of people	Fernandez et al. (2012); Kross et al. (2013); Ortigosa et al. (2014)
Epidemic of infection disease	Santillana et al. (2015)
Fake news	Gupta et al. (2013); Conroy et al. (2015)
Heart disease	Eichstaedt et al. (2015)
Mental health	De Choudhury et al. (2013); Guntuku et al. (2017)
Popularity of news	Bandari et al. (2012)
Movie ratings	Oghina et al. (2012)
Opinion flow	Wu et al. (2014)
Personal traits and characters	Kosinski et al. (2013)
Public health	Robillard et al. (2013); Sinnenberg et al. (2017)
Sharing cascades	Kupavskii et al. (2012); Cheng et al. (2014)
Stock market behavior	Bollen et al. (2011); Siganos et al. (2014)
Rainfall levels	Lampos and Cristianini (2012)
Suicide rates	Won et al. (2013)
Tourism	Zeng and Gerritsen (2014)
Word-of-mouth (WOM) or consumer reviews	Zhang et al. (2012)

As one can see from Table 1 there are many different questions studied so far. In order to organize these publications, we introduce a taxonomy to categorize these publications according to a few major variables. In Figure 1 we give a graphical summary of our taxonomy. Overall, these questions fall into seven different fields (E, Economy; G, Geophysics; H, Health; M, Management; S, Sociology; Ps, Psychology; Po, Politology) covering almost all science areas. In this figure, we provide furthermore information about four additional layers, namely (I) the time horizon of the prediction (horizon) for making predictions about the future (F) or the present (P), (II) the level of prediction (level) for macro (Ma) and micro (Mi) level predictions, (III) the time of prediction (time) for batch (Ba) and real-time (Rt) predictions, and for (IV) making spatial (Sp) or non-spatial (Ns) predictions. Each of these layers will be discussed in the following sections.

**Figure 2:**
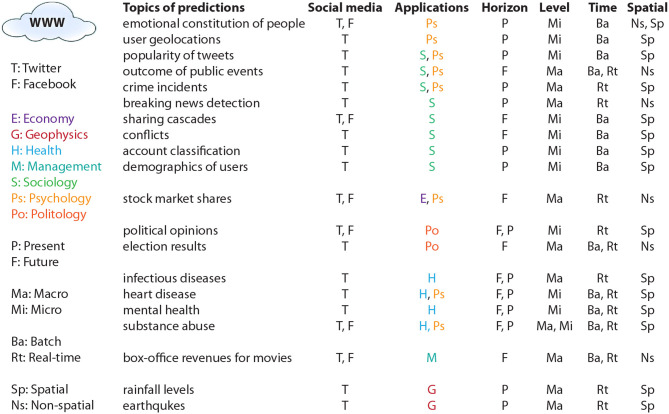
Taxonomy of questions that have been investigated so far by prediction models. Overall, these questions fall into seven different applications. E, Economy; G, Geophysics; H, Health; M, Management; S, Sociology; Ps, Psychology; Po, Politology. In addition, distinctions are made regarding the horizon, level, time, and spatial nature of the predictions (see main text for details).

One area missing from the above (see Figure 2) were studies in humanities. By performing a WoS search looking for articles containing the words Twitter/Facebook, humanities, and prediction/forecast we found no results. However, we found articles (54) searching for Twitter/Facebook and humanities. Interestingly, these articles are descriptive rather than predictive in nature. Examples for such studies are (Vainio and Holmberg, 2017). In Lee et al. (2017) and Vainio and Holmberg (2017) the authors studied who tweeted scientific articles with at least one Finnish author/co-author and that had high altmetric counts on Twitter and in Lee et al. (2017) the use of Twitter by scholars in the digital humanities was studied for informal scholarly communication. Those and similar papers performed a descriptive statistical analysis but no predictions were made.

### 3.2. Time horizon of the forecasting

There are two different types of prediction models used in the literature with respect to the prediction itself. The first type predicts the future and the second prediction type predicts the present. The former type is naturally understood because this is what is usually implied by a prediction or a forecast, namely that it should tell us something about the near or far future. For this reason, almost all of the above studies are from this type. However, the second type is unconventional because neither in classical statistics nor machine learning such predictions are made. An example in our context is the prediction of rainfall levels Lampos and Cristianini (2012). Here the idea is to use Twitter users as sort of *social sensors* that report real-world events instantaneously. Another example is the prediction of earthquakes (Sakaki et al., 2010). In the literature such predictions are called *nowcasting* or *predicting the present* (Schoen et al., 2013).

### 3.3. Macro- vs. micro-level predictions

Another distinction in the predictions is with respect to the level of the prediction. The majority of articles makes predictions on a macro-level for which individual Twitter or Facebook users are irrelevant. Instead, what is important is the aggregation of users into categories. Examples for this is, e.g., predicting outcome of elections or box-office success of movies (Asur and Huberman, 2010; Alonso and Vilares, 2016; Tumasjan et al., 2011). In contrast, predictions on the micro-level make predictions for Twitter or Facebook users themselves. Examples are predicting the personality (Golbeck et al., 2011; Quercia et al., 2011; Hughes et al., 2012; Youyou et al., 2015) or human mobility (Jurdak et al., 2015).

### 3.4. Batch vs. real-time predictions

The difference between batch and real-time models is that in the former case data are gathered off-line and then one prediction is made. In the latter case this process is iterated multiple times and data are generated on-line. Examples for batch predictions are election forecasts whereas real-time predictions forecast the political opinion continuously (Alonso and Vilares, 2016; Tumasjan et al., 2011). In general, the need for developing a real-time model depends on the application one is aiming at. For instance, if one intends to predict the outbreak of an epidemic of an infection disease this needs to be done in a real-time manner because there is not one scheduled event to occur one wants to predict but there is all the time a possibility for the outbreak to happen (Robillard et al., 2013; Santillana et al., 2015). Another example is the prediction of stock market values (Bollen et al., 2011; Siganos et al., 2014).

### 3.5. Non-spatial vs. spatial predictions

A final distinction of prediction models relates to non-spatial vs spatial predictions. A non-spatial prediction makes a forecast for the population as a whole, e.g., the outcome of an election (Alonso and Vilares, 2016; Tumasjan et al., 2011). In contrast, a spatial prediction makes a forecast for, e.g., all municipalities of a country. In this sense predictions in the former case can be considered as *scalar* whereas in the latter case they are *multivariate*. In order to accomplish a spatial prediction, usually information about the geolocation of the users is utilized. This information may be either directly available, or needs to be inferred from the content of the microblogs.

## 4. Discussion

As we have shown in Figure 1B, the interest in studying data from social media increases every year. However, also the proportion of prediction related publications increases every year. In order to see this we show Figure 1C. In this scatter plot we show results we obtained from a WoS search for articles containing the social media (SM) keyword Twitter or Facebook (x-axis) and for articles containing additionally the keywords “prediction” or “forecast” (y-axis). The fraction of the values on the y-axis to the values on the x-axis, i.e., *y*_*i*_/*x*_*i*_, gives the percentage of prediction related publications compared to all publications. In Figure 1C. the shows values are for Twitter (values for Facebook are similar). Due to the fact that the number of publications increases every year, as can be seen from Figure 1B, the x-axis in this figure is proportional to the publication year and, hence, one can see that the fraction of prediction related publications increases over the years reaching currently well over 60%.

### 4.1. Gaps in the literature

When collecting the articles for this review we noticed that despite the fact that all considered publications utilize prediction models, only a small fraction of these make an attempt to ensure the statistical soundness of the models. As a simple indicator for this omission we searched the WoS for articles containing the keywords Twitter or Facebook and for articles that contain the keywords Twitter and cross validation or Twitter and resampling (similarly for Facebook). The result of these searches is shown as a scatter plot in Figure 1D. The shown pairs correspond to the same publication year and y-axis label SM & CV is an abrieviation for our second search query. This figure confirms our perception indicating that only a small fraction of all articles applies resampling methods in order to quantify the uncertainty in the data and to guard against overfitting. Given the fact that the analyzed social media data are “big,” resampling methods can always be applied. Overall, this indicates a possible problem that would require further analysis.

### 4.2. Potential future developments

#### 4.2.1. Data integration

The vast majority of studies analyzed only data from social media. However, a combination of such data with external data would allow to address further questions. For instance, health related studies could benefit from *integrating data* from disease databases, e.g., Online Mendelian Inheritance in Man (OMIM) (OMI, 2007), Gene Ontology (Ashburner et al., 2000), or DrugBank (Wishart et al., 2007). This approach enables also in a natural way the extension of text mining approaches because the external information may be utilized in form of dictionaries, e.g., lists of words from a specific category, that can be used to perform a guided sentiment analysis.

Support for our argument for using external information is provided by Ciulla et al. (2012). The authors found that information provided by tweets alone is not sufficient in order to predict the outcome of a social event (the winner of American Idol) but tweets need to be complemented with information about the geographic location of the tweets.

Another purpose for data integration could be for increasing prediction accuracy and reducing prediction errors. This could be accomplished by utilizing different, independent sources of social media data. In this way one could also naturally obtain quantitative estimates for the variability in the data.

#### 4.2.2. Social networks

A further direction to explore could be the utilization of social networks (Wasserman and Faust, 1994). An example area where this could be of relevance is studies about infectious outbreaks. The reason for this is that an infection can only spread by human contacts. However, usually, this human contact network is not known. As an approximation for such a human contact network one could utilize data from social media to infer such a network. The simplest way to do this could be by utilizing the information “who is a follower of whom” which can be directly extracted from Twitter. However, one can go beyond these follower networks by also constructing semantic networks. The semantic networks could be constructed from estimating the similarity, e.g., among Twitter users based on the content of their tweets and conditioned on metadata. As a result, the information from these different networks could be integrated leading to characteristic spatial scores of the twitter activity and content in specific area.

#### 4.2.3. Deep learning

Finally, it will be interesting to see if new machine learning and artificial intelligence methods, above all deep learning methods (Hinton et al., 2006; Bengio et al., 2009; LeCun et al., 2015), e.g., deep neural networks, deep decision trees or deep belief networks, will change the *type of questions* addressed with social media data. So far, deep learning methods have found ample applications in image recognition, audio classification, genomics and text mining, e.g., (Lee et al., 2009; Alipanahi et al., 2015; Jiang et al., 2015; He et al., 2016), however, for social media mining we cannot observe from the current literature that new “What to predict” questions have emerged. Instead, familiar questions are studied with these new methodologies focusing on “How to predict.” Maybe, more experience is needed until scientists find new questions that can be raised with such computer- and data-intense approaches.

## 5. Conclusions

In this paper we surveyed the literature of prediction models for social media with a focus on the questions that have been addressed so far. Since we are observing a transition from descriptive to predictive studies in the last years (see Figure 1C) a taxonomy of such questions is a natural first step in understanding the capabilities of social media. We anticipate this trend to continue and the diversity of question to increase. However, a necessity for the latter is a better comprehension of the data social media provide by exploring their limitations and possibilities with respect to statistical models.

## Author contributions

All authors listed have made a substantial, direct and intellectual contribution to the work, and approved it for publication.

### Conflict of interest statement

The authors declare that the research was conducted in the absence of any commercial or financial relationships that could be construed as a potential conflict of interest.
